# GhABP19, a Novel Germin-Like Protein From *Gossypium hirsutum*, Plays an Important Role in the Regulation of Resistance to Verticillium and Fusarium Wilt Pathogens

**DOI:** 10.3389/fpls.2019.00583

**Published:** 2019-05-08

**Authors:** Yakun Pei, Xiancai Li, Yutao Zhu, Xiaoyang Ge, Yun Sun, Nana Liu, Yujiao Jia, Fuguang Li, Yuxia Hou

**Affiliations:** ^1^College of Science, China Agricultural University, Beijing, China; ^2^State Key Laboratory of Cotton Biology, Institute of Cotton Research, Chinese Academy of Agricultural Sciences, Anyang, China

**Keywords:** germin-like protein, *Gossypium hirsutum*, *Verticillium dahliae*, *Fusarium oxysporum*, superoxide dismutase, disease resistance

## Abstract

Germin-like proteins (GLPs) are water-soluble plant glycoproteins belonging to the cupin superfamily. The important role of GLPs in plant responses against various abiotic and biotic stresses, especially pathogens, is well validated. However, little is known about cotton GLPs in relation to fungal pathogens. Here, a novel GLP gene was isolated from *Gossypium hirsutum* and designated as *GhABP19*. The expression of *GhABP19* was upregulated in cotton plants inoculated with *Verticillium dahliae* and *Fusarium oxysporum* and in response to treatment with jasmonic acid (JA) but was suppressed in response to salicylic acid treatment. A relatively small transient increase in *GhABP19* was seen in H_2_O_2_ treated samples. The three-dimensional structure prediction of the GhABP19 protein indicated that the protein has three histidine and one glutamate residues responsible for metal ion binding and superoxide dismutase (SOD) activity. Purified recombinant GhABP19 exhibits SOD activity and could inhibit growth of *V. dahliae*, *F. oxysporum*, *Rhizoctonia solani*, *Botrytis cinerea*, and *Valsa mali in vitro*. To further verify the role of *GhABP19* in fungal resistance, *GhABP19*-overexpressing *Arabidopsis* plants and *GhABP19*-silenced cotton plants were developed. GhABP19-transgenic *Arabidopsis* lines showed much stronger resistance to *V. dahliae* and *F. oxysporum* infection than control (empty vector) plants did. On the contrary, silencing of *GhABP19* in cotton conferred enhanced susceptibility to fungal pathogens, which resulted in necrosis and wilt on leaves and vascular discoloration in *GhABP19*-silenced cotton plants. The H_2_O_2_ content and endogenous SOD activity were affected by *GhABP19* expression levels in *Arabidopsis* and cotton plants after inoculation with *V. dahliae* and *F. oxysporum*, respectively. Furthermore, *GhABP19* overexpression or silencing resulted in activation or suppression of JA-mediated signaling, respectively. Thus, GhABP19 plays important roles in the regulation of resistance to verticillium and fusarium wilt in plants. These modulatory roles were exerted by its SOD activity and ability to activate the JA pathway. All results suggest that *GhABP19* was involved in plant disease resistance.

## Introduction

Germin-like proteins (GLPs) are diverse and ubiquitous plant glycoproteins, ordinarily found in various terrestrial plants (Rebeccam et al., 2009). GLPs belong to the cupins, a functionally diverse superfamily of proteins that contains a conserved β-barrel core, which is involved in manganese ion binding (Dunwell et al., 2008). Previous studies have indicated that GLPs are stable in heat, extreme pH, and detergent treatments (Woo et al., 2000), and they can function as various enzymes, such as superoxide dismutase (SOD; Banerjee and Maiti, 2010; Rietz et al., 2012; Zhang et al., 2017), oxalate oxidase (OXO; Sakamoto et al., 2015), and ADP glucose pyrophosphatase/phosphodiesterase (Fan et al., 2005), polyphenol oxidase (Cheng et al., 2014), as well as rhicadhesin receptors (Swart et al., 1994).

Based on plant-microbe interactions, GLPs are considered a pathogenesis-related (PR) protein 16 family (Park et al., 2004). Many GLPs are localized in the cell wall and function as cofactors for cell wall reinforcement by facilitating the cross-linking of plant cell wall components during the formation of papillae, which renders them resistant to infection; this process involves the generation of H_2_O_2_ because of their SOD or OXO activity (Wei et al., 1998; Liu Q. et al., 2016; Zhang et al., 2017). SOD is responsible for the dismutation of superoxide to H_2_O_2_ and O_2_ and the protection of cells from oxidative burst (Sultana et al., 2016). H_2_O_2_ can act as a signaling molecule for the induction of systemic acquired resistance of non-inoculated tissues (Tran et al., 2014). In addition, H_2_O_2_ can initiate salicylic acid (SA) and/or jasmonic acid (JA) signaling pathways, leading to the synthesis of pathogenesis-related protein synthesis and plant defenses, respectively (Leon et al., 1995).

An increasing amount of evidence has shown that GLPs are a crucial component of plant basal host resistance and can be upregulated and/or activated by pathogen infection or by the application of disease resistance–associated chemicals such as H_2_O_2_, SA, and ethylene (Lou and Baldwin, 2006; Zimmermann, 2006; Godfrey et al., 2007; Himmelbach et al., 2010). The heterologous expression of GLPs or gene silencing of endogenous GLPs have also provided evidence for their involvement in defense against fungal pathogens. For example, the transient overexpression of *HvGER4* and *HvGER5* or silencing of *HvGER3* protect barley epidermal cells from *Blumeria graminis* infection (Zimmermann, 2006). Moreover, the heterologous expression of *BvGLP-1* from sugar beet (*Beta vulgaris*) increased the resistance of transgenic *Arabidopsis thaliana* against *Verticillium longisporum* and *Rhizoctonia solani* infection (Knecht et al., 2010). In *Lilium regale*, the *LrGLP1* gene could be induced by exogenous ethylene and the incompatible interaction between *L. regale* and *Fusarium oxysporum* f. sp. *lilii*; the *LrGLP1* transgenic tobacco lines showed considerably stronger resistance to *F. oxysporum* f. sp. *lilii* infection than did the wild-type plants (Zhang et al., 2017). Furthermore, the *OsRGLP1* gene was highly expressed in *Medicago truncatula* transformed lines and provided protection against *F. oxysporum* (Sultana et al., 2016).

Verticillium and fusarium wilt, caused by *Verticillium dahliae* and *F. oxysporum*, respectively, are two important fungal diseases of cotton that affect the vascular tissues (Ma et al., 1997; Zambounis et al., 2008). It is extremely difficult to control verticillium wilt in cotton, as its hyphae reside in the woody vascular tissues and thus cannot be destroyed by fungicides. In addition, *V. dahliae* survives in soil for many years because of the production of microsclerotia (Fradin and Thomma, 2006). *F. oxysporum* is widespread and pathogenic to various plant species. It is able to grow and survive over long periods on organic matter in soil and in the rhizosphere of many plants. This fungus penetrates the roots or invades the vascular system of host plants, causing either root-rot or tracheomycosis (Fravel et al., 2003). A series of resistance-related genes from cotton have been studied to uncover the resistance mechanisms of cotton against *V. dahliae* and *F. oxysporum* (Liu et al., 2017b; Li et al., 2018; Wang et al., 2018). However, data on the functions of cotton GLPs in fungal defense are still poorly understood. Therefore, assessing the biochemical properties of GhABP19 and identifying its functions in cotton disease resistance is necessary.

Previous studies have shown that two homologous auxin-binding proteins, PpABP19 and PpABP20, isolated from shoot apices of *Prunus persica*, are highly homologous to families of germin and GLPs and belong to the GLP family (Ohmiya et al., 1998; Ohmiya, 2002). In the present study, a novel *ABP19* gene was isolated from *Gossypium hirsutum*. The expression patterns of *GhABP19* that can be induced by pathogen infection, abiotic stresses, and phytohormones were characterized in cotton. We generated a recombinant GhABP19 protein by expressing it in *Escherichia coli* and evaluated its role in the defense against fungal infections and its SOD activity. GhABP19 is involved in the defense against *V. dahliae* and *F. oxysporum* infection; the results were examined by generating *GhABP19*-overexpressing *Arabidopsis* plants and *GhABP19*-silenced cotton plants through virus-induced gene silencing (VIGS) assays. Further, we showed that *GhABP19* is involved in the JA-mediated defense response.

## Materials and Methods

### Plant Materials and Fungal Strains

The resistant cotton cultivar Zhongzhimian 2 (original strain GK44) and the susceptible cultivar Xinluzao 33 provided by the Cotton Research Institute were grown under standard conditions, and their seedlings were used in this study. The seeds of *A. thaliana* (ecotype Columbia) were sterilized, rinsed and then sowed on Murashige-Skoog (MS) culture medium. After 4°C verbalization for 3 days, seeds were cultured in chamber (22°C/18°C, 16 h light/8 h dark) and 10-days-old seedlings were transplanted into soil. Virulent strains of *V. dahliae* (strain Vd991) and *F. oxysporum* (strain AYF-1) were grown in the potato dextrose agar (PDA) at 25°C for a week. Colonies were then transferred to 500-mL Erlenmeyer flasks containing 100 mL Czapek’s liquid medium and grown at 25°C for 7 days. Concentrated spore solutions were then prepared using 10^7^-conidia/mL suspensions.

### Cloning of *ABP19* cDNA

Total RNA of Zhongzhimian 2 cotton cultivar complete stool (grew under standard conditions) was extracted using a commercially available kit (Promega, Madison, WI, United States). Polyadenylated mRNA was separated with a PolyATract mRNA Isolation System (Promega, Madison, WI, United States). Subsequently, a cDNA library was generated by inserting fragments in a λZAP-II vector as the specifications of cDNA Library Construction Kit (Merck, Germany). The library was propagated on 140 mm plates to obtain about 10^5^ plaques (Wang et al., 2011a). The conserved region of *AtGLP3a* isolated from *Arabidopsis* (Staiger et al., 1999), was labeled with ^32^P-dUTP and used as probe for positive plaques by colony *in situ* hybridization. A positive plaque was obtained after three rounds, and the fragment was subcloned into pBlueScript II SK (+) through *in vivo* excision, following the protocol provided by manufacturer (Stratagene, United States).

### Bioinformatic Analyses of *GhABP19*

The open reading frame (ORF) of *GhABP19* was identified using the program ORF Finder at the NCBI web-site^1^. The prediction of the putative signal peptide sequence was done at the SignalP 4.1 server^2^. Theoretical molecular mass and isoelectric point (pI) were assessed through the ProtParam website^3^. Post-translationa modifications as *N*-acetylation and phosphorylation were predicted through NetAcet 1.0 and NetPhos 2.0 server, respectively (Blom et al., 2004; Kiemer et al., 2005). Multiple sequence alignment was conducted with Clustal Omega^4^ under default settings. The phylogenetic tree was performed by MEGA 7.0 software using the neighbor-joining method (Kumar et al., 2016). The Genbank accession numbers of germin and germin-like protein sequences have been presented in Supplementary Table S1.

### Expression Analysis of the *GhABP19*

For pathogen treatment, roots of 2-week-old cotton seedlings were inoculated with *V. dahliae* and *F. oxysporum* conidial suspension (both of 10^7^-conidia/mL) for 5 min, respectively, then transplanted into pots with fresh soil. For abiotic stresses and hormone treatments, cotton seedlings of true leaf stage were uprooted from soil and replanted in Hoagland medium, which contained 100 mM H_2_O_2_, 1 mM SA and 100 μM JA, respectively. The leaf tissues from cotton plants were harvested at an appropriate time for RNA extraction. Total RNA was isolated using an EASYspin RNA Extraction Kit (Biomed, China) from cotton tissues. First-strand cDNA was synthesized by Fast Quant cDNA Reverse Kit (TIANGEN BIOTECH CO., LTD.), diluted, and used as template for real-time quantitative reverse transcription-polymerase chain reaction (qRT-PCR). *GhABP19* was amplified by primer sequences 5′-CAACGCAGCCGACTTCTGTG-3′ and 5′-CCCAGGGTGTGTATGATAGG-3′. The endogenous gene *UBQ7* (DQ116441) from cotton was used as an internal standard, and can be detected using the primers 5′-GACCCTTCCTCTATATAAG-3′ and 5′-GGACAACTCCATGAAAAG-3′. Reactions were prepared in 20 μL with SYBR Premix Ex Taq (Tli RNaseH Plus; Takara, Shiga, Japan) and amplification was performed on an ABI 7500 thermocycler (Applied Biosystems, Foster city, CA, United States). qRT-PCR assays were carried out in three independent biological samples per treatment and three technical replicates per samples. Expression was calculated with 2^-ΔΔCT^ method and data were analyzed in Origin 8.

### Expression and Purification of Recombinant GhABP19 Protein

GhABP19 was amplified using primers, which included restriction sites for *Nde* I and *Hind* III enzymes in the forward and reverse ones, respectively. The sequence of these primers (restriction sites are underlined) were as follows: (Forward)5′-GGACCATATGATGGACATACCTTCAAGGAC-3′, (Reverse) 5′-ACCCAAGCTT ACCAGTTCCTCCAAGAACA-3′. The pET-22b(+) protein expression vector (Novagen) was also digested with *Nde* I and *Hind* III and then ligated with the *GhABP19* fragment by using T4 ligase (Promega) for overnight at 16°C; the resulting construct was named as pET-22b(+)-*GhABP19*. Next, the construct was transformed into *E. coli* BL21 (DE3), and single colonies were cultured at 37°C in Luria–Bertani broth supplemented with 100 μg/mL ampicillin. The cultures were scaled up by inoculating precultures at 1% into fresh media. At OD 0.6–0.7, they were immediately induced with 0.1 mM IPTG and cultured for another 4 h at 37°C with oscillation. Samples were harvested by centrifugation for 10 min at 10,000 ×*g* and then resuspended in 20 mL phosphate buffered saline (PBS, pH 7.4). Cells were disrupted using a sonicator (Ultrasonic processor), and then centrifuged at 10,000 ×*g* for 10 min. Inclusion fraction was separated and resuspended in PBS. Protein was verified using sodium dodecyl sulfate polyacrylamide gel electrophoresis (SDS-PAGE), and GhABP19 purification was performed using 6 × His-Tagged Protein Purification Kit (CW BIO) following manufacturer’s recommendations.

### Antifungal Activity Assay of Purified GhABP19 Protein

The fungal species *V. dahliae*, *F. oxysporum*, *R. solani*, *B. cinerea*, and *V. mali* were grown in PDA plates at 25°C. Five different concentrations (35, 70, 140, 280, and 560 μg/mL) of GhABP19 were added in PDA plates at 25°C, then a small amount of mycelia (0.5 cm in diameter) was placed on the surface of the agar. The half maximal inhibitory concentration (IC_50_) of pure protein was determined as previous described (Wang et al., 2011b). Data were collected from three independent replicates.

### Expression Vector Constructs and Generation of *Arabidopsis* Transgenic Plants

Plant expression vector Super-pCAMBIA1300 was used in the present study. The ORF of *GhABP19* was amplified using the forward primer 5′-CTAGTCTAGAATGGACATACCTTCAAGGAC-3′ and reverse primer 5′-AAGGACTAGTACCAGTTCCTCCAAGAACA-3′, which restriction sites *Xba* I and *Spe* I were, respectively, added (restriction sites are underlined). Then, the gene was ligated into the digested Super-pCAMBIA1300 vector with the same two restriction endonucleases for fusion with C-terminal green fluorescent protein gene (*GhABP19-GFP*). The vector expressed *GhABP19* under control of the CaMV35S promoter and carried the hygromycin phosphotransferase (*htp* II) resistance gene as a selectable marker for the selection of transformed plants (Supplementary Figure S1A). The plasmid was introduced into *Agrobacterium tumefaciens* strain GV3101 by freeze–thaw method. *Arabidopsis* plants were transformed by the floral dip method via *Agrobacterium*-mediated transformation procedure (Clough and Bent, 1998), control plants were transformed with Super1300-GFP empty vector plasmid.

### Infection Assay of *Arabidopsis* Plants With Fungal Conidia in Soil

Antifungal activities induced by *GhABP19* in *Arabidopsis* plants after *V. dahliae* and *F. oxysporum* infection were assessed using the method described before (Stoilova and Chavdarov, 2006) with slight modification. Seedlings of *Arabidopsis* plants were cultivated in soil in small pots for 4 weeks under normal growth condition. When the spore concentrations of *V. dahliae* and *F. oxysporum* reached 10^7^ conidia/mL, the susceptibility of *Arabidopsis* to these isolates was determined by dipping the roots of 4-week-old *GhABP19*-transgenic and control (empty vector) plants in fungal culture for 5 min after wounding four to five roots on each plant by trimming before planting. The same number of plants injured and dipped in sterilized water served as non-infected controls. The symptoms of *V. dahliae* and *F. oxysporum* infection on *Arabidopsis* plant leaves in the form of yellowing, wilting, and drying were recorded for 30 and 49 days, respectively. Severity of infection on the plants from mild to severe was recorded on a scale of 1–5, indicating no symptoms, greenish yellow leaves, yellow leaves, mildly wilted leaves, and wilted and completely dried leaves, respectively. The numbers of leaves in each grade were recorded. Percent disease index (PDI) was calculated using the following formula:

$$\text{PDI}=\frac{0\times (\text{S}1)+1\times (\text{S}2)+2\times (\text{S}3)+3\times (\text{S4})+4\times (\text{S5})}{\text{Total number of leaves}\times \text{maximum scale}}\times 100$$

where S1–S5 stand for the number of leaves in each scale. Morphological and growth data on infected transgenic plants were compared with those of infected control plants. Data were collected from three independent replicates (*n* ≥ 30), and similar results were obtained.

### Roots Staining Assays and Light Microscopy Observations

To monitor fungal hyphae on or in *Arabidopsis* roots, seedlings were removed from agar plates, roots of control and transgenic *Arabidopsis* plants were washed thoroughly in running tap water, cut into 1-cm pieces and treated overnight with 10% KOH solution at room temperature. Thereafter, the root pieces were washed 3–5 times with sterilized distilled water and treated with 1% HCl for 3–4 min before staining with 0.05% trypan blue in lactophenol. The stained root segments were examined microscopically. The assays were repeated three times (*n* ≥ 20), and similar results were obtained.

### Virus-Induced Gene Silencing (VIGS)

Silenced fragments of *GhCLA1* (*cloroplastos alterados 1*) and *GhABP19* were cloned from Zhongzhimian 2 cotton cultivar cDNA. In detail, the *GhCLA1*-silenced fragment (500 bp) was cloned using the primers 5′-GGAATTCCACAACATCGATGATTTAG-3′, and 5′-GGGGTACCATGATGAGTAGATTGCAC-3′, according to previous study (Gao et al., 2011). The *GhABP19*-silenced fragment (457 bp) was amplified using the primers 5′-CGACGACAAGACCGTGACCATGAAATGACTTCGTTTACTCCGGCC-3′ and 5′-GAGGAGAAGAGCCGTCATTAGCTTCTTAATCTGAGCAGCATCCAG-3′. In a subsequent step, both of the silenced fragments were inserted into the *TRV:00* vector by ligation-independent cloning to generate the *TRV:GhCLA1* and *TRV:GhABP19* vectors. The plasmids of *TRV1*, *TRV:GhCLA1*, and *TRV:GhABP19* were transformed into *A. tumefaciens* strain GV3101 by heat shock. Then, *A. tumefaciens* containing *TRV1* and *A. tumefaciens* containing *TRV:GhABP19* or *TRV:00* were mixed in equal amounts and injected into the cotyledons of 2-weeks-old Zhongzhimian 2 cotton cultivar seedlings to generate the *GhABP19-*silenced (*TRV:GhABP19*) cotton and control (*TRV:00*) cotton plants. TRV:GhCLA1 plants were used as positive controls as described previously (Gao et al., 2011) and showed clear signs of albinism in leaves after VIGS for 2 weeks (Supplementary Figure S2). TRV:GhABP19 and *TRV:00* would be challenged with *V. dahliae* and *F. oxysporum* by syringe inoculation according to Liu N. et al. (2016). The assays were repeated three times (*n* ≥ 20), and similar results were obtained.

### Measurement of H_2_O_2_ Content and SOD Activity

All *Arabidopsis* and Zhongzhimian 2 cotton cultivar were inoculated with two drops (5 μL) of *V. dahliae* and *F. oxysporum* conidial suspension per leaf (both of 10^7^-conidia/mL; four leaves per plant in *Arabidopsis* and on the true leaves of cotton), respectively. Infected leaves were collected at 48 h post inoculation (hpi) and as samples for H_2_O_2_ content and SOD activity measurement. FOX reagent was performed to measurement the content of H_2_O_2_ in samples under normal or inoculation conditions (Cheeseman, 2006). Samples were ground with liquid nitrogen and H_2_O_2_ was extracted by adding HCl (400 μL, 25 mM) to samples, centrifuged. 100 μL supernatant was added to 900 μL FOX reagent [ammonium iron (II) sulfate (250 μM), sorbitol (100 μM), xylenol orange (100 μM), H2SO4 (25 mM), 1% ethanol] and incubated for 30 min at 30°C, A_560_ was measured. The total SOD activity of purified protein and endogenous SOD activity of samples were measured using a SOD assay kit (Beyotime Institute of Biotechnology, Jiangsu, China) with WST-8 method. Commercial bovine erythrocyte Cu/Zn-SOD was used as positive control (Acmec Biochemical, Shanghai, China). The assays were repeated three times (*n* ≥ 20), and similar results were obtained.

### Expression Level of Defense-Related Genes in *Arabidopsis* and Cotton Plants

Total RNA was extracted from samples obtained from non-inoculated leaves with TRNzol RNA kit [TIANGEN BIOTECH (Beijing) CO., LTD.]. The procedures of synthesizing the first-strand cDNA and qRT-PCR assays were preformed just as mentioned before. The *Arabidopsis* housekeeping gene *elongation factor 1*α (*EF1*α) and *GhUBQ7* were employed as internal standards. The relative expression was determined by the 2^-ΔΔCT^ method and data were analyzed in Origin 8. qRT-PCR assays were carried out in three independent biological samples per treatment and three technical replicates per samples. Primers used for this analysis are shown in Supplementary Table S2.

### Homology Modeling

Initial homology model of GhABP19 was generated using SWISS-MODEL workspace^5^ (Bordoli et al., 2009). The crystal structure of *Hordeum vulgare* germin (2ET7) was used as template to predict the theoretical model. All three-dimensional models were analyzed and visualized using EzMol, Version 1.20^6^ (Reynolds et al., 2018).

### *Cis-*Elements Analysis for the Promoter

1500 bp sequence upstream of *GhABP19* was downloaded from NCBI for *cis*-elements analysis. The sequence was scanned by PLACE^7^ which is a database of nucleotide sequence motifs found in plant *cis*-acting regulatory DNA elements (Higo et al., 1999).

### Statistical Analysis

All experiments and measurements were performed with three replicates per treatment. Analysis of variance (ANOVE) was carried out using statistical software IBM SPSS statistics 20. Data are presented as mean ± standard error. Significant differences were determined at the 5 and 1% level of significance and asterisks are used to indicate *p*-values: ^∗^*p* < 0.05, ^∗∗^*p* < 0.01.

## Results

### Characterization and Sequence Analysis of *GhABP19*

**FIGURE 1:**
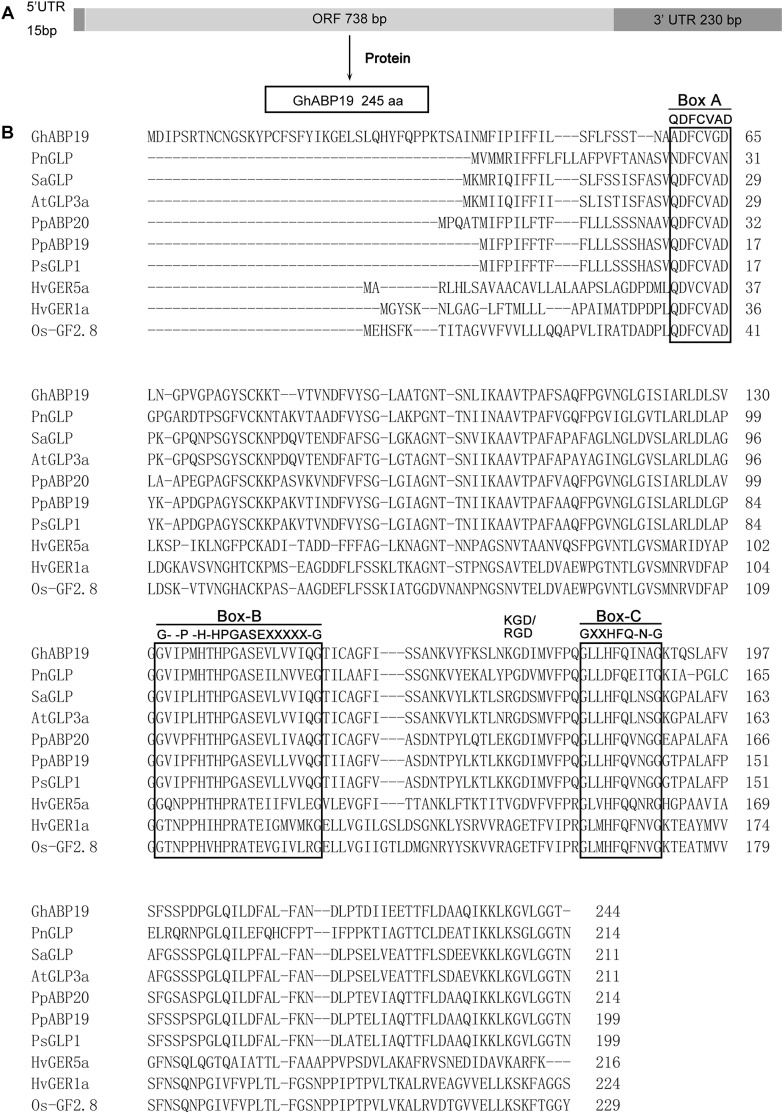
Sequence analysis of *GhABP19* and multiple sequence alignment of germins and germin-like proteins. **(A)** Schematic representation of GhABP19 primary transcript. **(B)** Typical motifs (boxes A, B, and C) are boxed. X indicates any hydrophobic amino acid. GenBank accession numbers for the individual sequences used in this analysis are listed in Supplementary Table S1.

*GhABP19* cDNA was obtained from cotton by using colony *in situ* hybridization, (GenBank accession number: MH430583). The full length cDNA of *GhABP19* is 983 bp, including 15 bp 5′ and 233 bp 3′ untranslated regions. The 735 bp predicted open reading frame encoded a protein of 244 amino acids without a signal peptide (Figure 1A). Multiple alignments of the *GhABP19* sequence with other reported true germin/GLP genes highlighted the numbers of conserved motifs and structural similarities that are common to the plant GLP subfamily. The predicted protein contains a KGD motif and three highly conserved germin/GLP oligopeptides, named boxes A, B and C (Figure 1B; Bernier and Berna, 2001).

The putative phosphorylation sites in GhABP19 were predicted using bioinformatic analysis. There are twelve sites at serine residues (positions 18, 26, 37, 50, 76, 89, 98, 122, 168, 193, 200, and 201) and six at threonine residues (positions 80, 82, 191, 220, 226, and 227). Serine at position 200 and threonine at position 227 have the likelihood of phosphorylation since their prediction scores were higher than those of the other potential phosphorylation sites (Blom et al., 2004). The prediction for *N*-acetylation was negative as no alanine, glycine, serine, or threonine was present at positions 1–3 in the amino acid sequence of GhABP19. The theoretical pI of the protein was 6.88 with a molecular mass of 25,922.88 Da. The instability index was 26.49, which suggested that the protein was stable, whereas the aliphatic index was 92.75 (Ikai, 1980).

**FIGURE 2:**
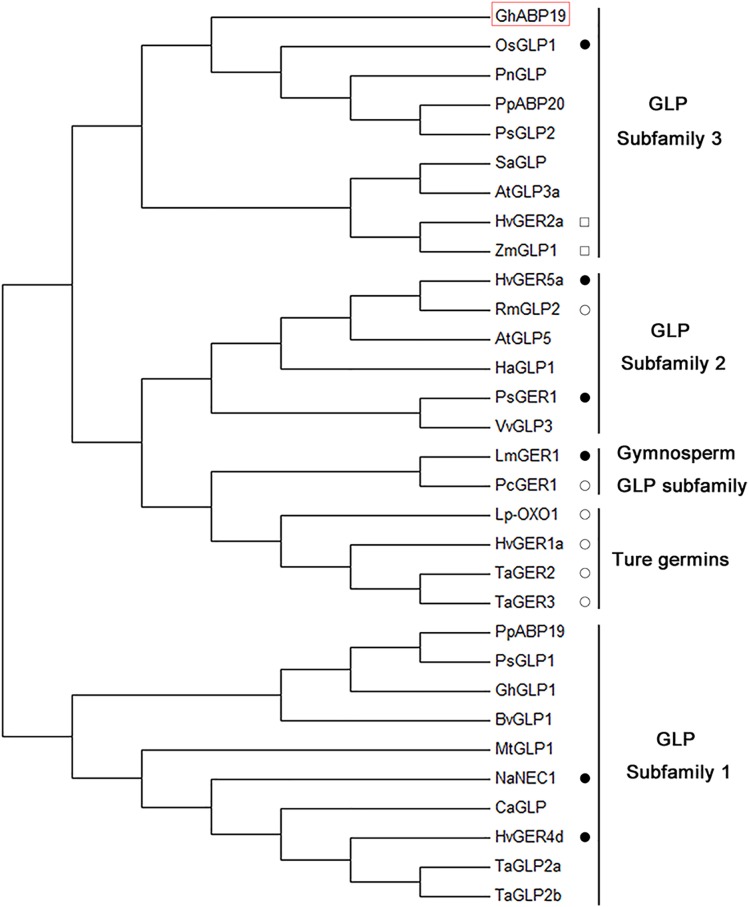
Phylogenetic analysis of GhABP19 together with other plant germins and germin-like proteins. The analysis involved 31 proteins; amino acid sequence of GhABP19 is in red box. Those proteins that have been analyzed for enzymatic activities are marked. Closed circles indicate the protein has superoxide dismutase activity. Open circles indicate that the protein has oxalate oxidase activity. Squares indicate that the protein has ADP glucose pyrophosphatase/phosphodiesterase activity. Sequences were aligned using MEGA 7.0 and the evolutionary history was inferred using the neighbor-joining method.

A phylogenetic tree comprising 31 germin/GLP sequences from 19 species was generated (Figure 2 and Supplementary Table S1). The dendrogram analysis revealed that germins/GLPs could be divided into five subfamilies, namely the true germin subfamily, GLP subfamily 1, GLP subfamily 2, GLP subfamily 3, and gymnosperm GLP subfamily (Carter and Thornburg, 2000). GhABP19 is a member of GLP subfamily 3, which is a very heterogeneous group. This group includes low-affinity auxin-binding proteins, such as PpABP20 form peach (Ohmiya et al., 1998) and PsGLP2 form *Prunus salicina*(El-Sharkawy et al., 2010); as well as GLPs known to express in a circadian pattern in *Pharbitis nil* (Ono et al., 1996), *Sinapis alba* (Heintzen et al., 1994), *A. thaliana* (Staiger et al., 1999), *H. vulgare* (Vallelian-Bindschedler et al., 1998), and *Zea mays* (Fan et al., 2005); and a disease resistance related protein OsGLP1 (Banerjee and Maiti, 2010).

### Expression Profiling of *GhABP19* in Cotton Cultivars

**FIGURE 3:**
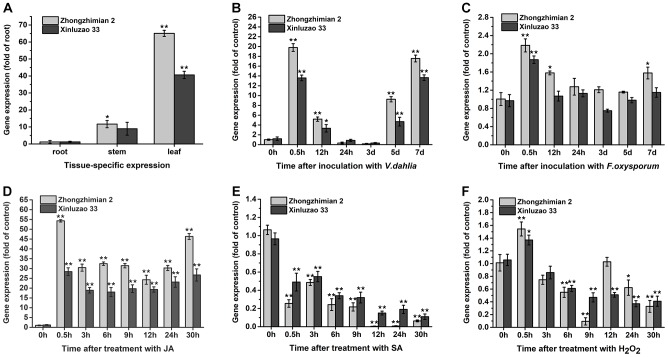
The expression patterns analysis of *GhABP19* gene under different conditions in the resistant Zhongzhimian 2 and the susceptible Xinluzao 33 cotton cultivars. **(A)** Tissue-specific expression of *GhABP19* gene. **(B,C)** *GhABP19* expression 0, 0.5, 12 and 24 h, 3, 5, and 7 days after inoculation with *Verticillium dahliae* **(B)** and *Fusarium oxysporum* **(C)**, control plants were treated with water. **(D–F)** *GhABP19* expression 0, 0.5, 3, 6, 12, 24, and 30 h after treatment with JA **(D)**, SA **(E)**, and H_2_O_2_ **(F)**, control plants were treated with Hoagland medium. *GhABP19* expression was quantified by qRT-PCR and compared to controls. Data were collected from three independent biological samples per treatment and three technical replicates per samples. Error bars represent standard error. Asterisks indicate a significant difference compared with control (^∗^*P* < 0.05, ^∗∗^*P* < 0.01, Student’s *t*-test).

We used qRT-PCR analysis to determine the *GhABP19* tissue-specific expression pattern in the resistant Zhongzhimian 2 and the susceptible Xinluzao 33 cotton cultivars, respectively. Root, stem, and leaf tissues were collected from both cotton cultivars under normal growth conditions, respectively. The results in Figure 3A indicated that *GhABP19* in Zhongzhimian 2 was preferentially expressed in leaf and stem tissue, while *GhABP19* only with high level of expression observed in leaf tissue in Xinluzao 33. Furthermore, these two cultivars were used to evaluate whether *GhABP19* expression was regulated in response to infection with *V. dahliae* or *F. oxysporum.* As shown in Figure 3B,C, *GhABP19* expression in Zhongzhimian 2 was significantly increased after inoculation with either *V. dahliae* and *F. oxysporum* for 0.5 h, 12 h, 5 days, 7 days or 0.5 h, 12 h, 7 days, respectively. The *GhABP19* transcription abundance in Xinluzao 33 also can be induced by pathogen infection, but its lower than that in Zhongzhimian 2. Next, JA and SA were applied to analyze whether *GhABP19* expression was related to phytohormone signaling. In both cotton cultivars, *GhABP19* transcription was immediately up-regulated following JA treatment; it was maintained at a high level until 30 h post treatment (Figure 3D). In contrast, *GhABP19* showed significantly reduced by SA stimulation (Figure 3E). In the presence of H_2_O_2_, *GhABP19* level was slightly increased at 0.5 h in both cotton cultivars and with a minimum level observed at 9 h in Zhongzhimian 2 and 24 h in Xinluzao 33, respectively (Figure 3F).

### Antifungal Activity of Purified Recombinant GhABP19 Protein

**FIGURE 4:**
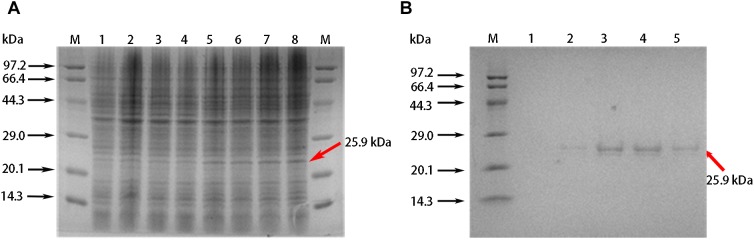
Purification of recombination GhABP19 protein. **(A)** SDS-PAGE gel electrophoresis for prokaryotic expression of GhABP19. Expressed proteins are indicated in arrows. Lane M, protein molecular weight markers. Lanes 1, 2, 3, and 4, crude extract without IPTG induction, and cultured at 37°C with oscillation for 2, 3, 4, and 5 h, respectively; lanes 5, 6, 7, 8, crude extract with 0.1 mM IPTG induction, and cultured at 37°C with oscillation for 2, 3, 4, and 5 h, respectively. **(B)** SDS-PAGE of purified recombination GhABP19. Lane M, protein molecular weight markers. Lane 1–5, purifier protein with different elution times.

We conducted a more detailed functional analysis of GhABP19 and produced the recombinant protein in the *E. coli* system. A protein with the expected molecular weight of the GhABP19 protein (25.9 kDa) was seen by SDS-PAGE after 2–5 h induction with 0.1 mM IPTG (Figure 4A). After the recombinant protein was purified using Ni columns, the elution fractions migrated as doublets on SDS gels (Figure 4B), the difference between the isoforms can be explained by the nature of germin glycan moieties (Jaikaran et al., 1990; Lane, 1994).

To investigate the antifungal activity of recombinant GhABP19 *in vitro*, we used a hyphal extension inhibition assay to measure the inhibitor activity of GhABP19 in response to pathogens such as *V. dahliae*, *F. oxysporum*, *R. solani*, *B. cinerea*, and *V. mali*. Results indicated that GhABP19 inhibited *F. oxysporum* and *V. dahliae* growth with IC_50_ 246.58 and 298.19 μg/mL, respectively (Table 1).

### SOD Structural Domain in GhABP19 and Its SOD Activity

**FIGURE 5:**
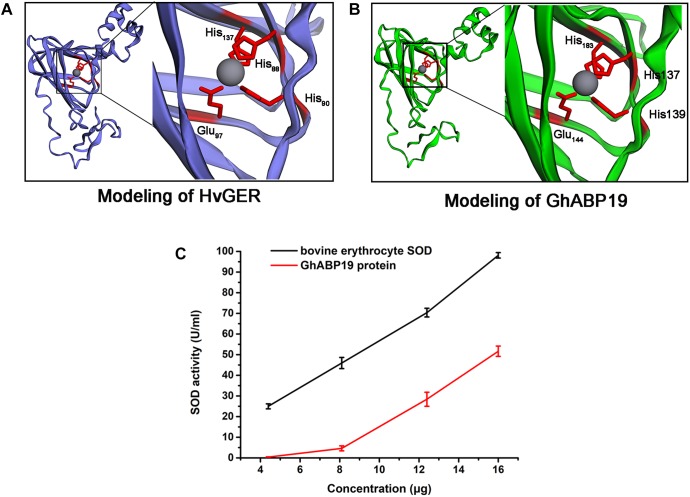
Structure predictions of GhABP19 and its SOD activity. **(A,B)** Modelings of *Hordeum vulgare* germin protein **(A)** and GhABP19 protein **(B)**. The *H. vulgare* germin amino acids–His88, His90, His137, and Glu97 and GhABP19 amino acids–His137, His139, His183, and Glu144 responsible for SOD domain are highlighted. **(C)** SOD activity of purified GhABP19 protein. 4.4, 8.1, 12.4, and 16.0 μg of purified GhABP19 protein was used in this assay. Commercial bovine erythrocyte Cu/Zn-SOD was used as positive control. Error bars represent standard error and shown for three replicates.

To understand the antifungal mechanism of GhABP19, we generated a three-dimensional model of this protein via homology modeling to investigate the presence of a domain to perform the function of fungal resistance. Barley germin (PDB number: 2ET7) was used as a template (Figure 5A) and showed 40.22% similarity to GhABP19. The GhABP19 model predicted active sites composed of three histidine (His137, His139, and His193) and one glutamate (Glu144) residues responsible for metal ion binding and SOD activity (Figure 5B). The active sites, analogous to barley germin, are protected within the jellyroll β-barrel structures, which are characteristic of the cupin superfamily.

**Table 1:** GhABP19 IC_50_ against fungal pathogens.

Pathogen	IC_50_ (μg/mL)
*Valsa mali*	65.78
*Rhizoctonia solani*	75.42
*Botrytis cinerea*	133.59
*Fusarium oxysporum*	246.58
*Verticillium dahliae*	298.19

*Five concentrations (35, 70, 140, 280, and 560 μg/mL) of purified GhABP19 were used for determination the IC_50_ of antifungal activity. Buffer was used as a control.*

Furthermore, we experimentally identified the SOD activity of the recombinant GhABP19 protein, which was predicated by structural features. SOD generates H_2_O_2_ and O_2_ by converting the superoxide radical anion (O_2_^-^). Thus, the most crucial procedure for determining the enzymatic ability of SOD is based on superoxide anion–dependent inhibiting reactions. The SOD activity was evaluated using a SOD assay kit (Beyotime Institute of Biotechnology, Jiangsu, China), in which WST-8 was used to produce a water-soluble formazan dye upon reduction with the superoxide anion generated by xanthine oxidase. SOD can inhibit this reaction by removing superoxide anion. The SOD activity could be measured using a colorimetric method. We used 4.4, 8.1, 12.4, and 16.0 μg GhABP19 protein to perform this assay (Figure 5C). Commercial bovine erythrocyte Cu/Zn-SOD was used as positive control (Acmec Biochemical, Shanghai, China).

### Subcellular Localization

**FIGURE 6:**
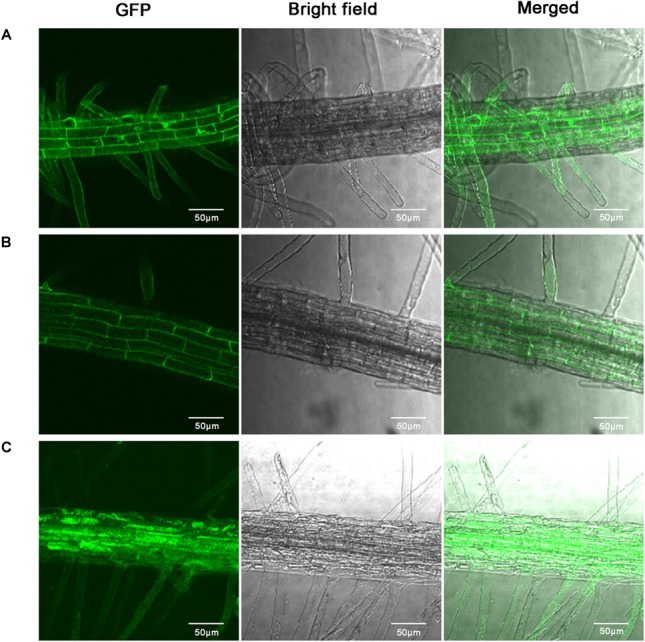
Subcellular localization of GhABP19-GFP fusion protein in *Arabidopsis* root cells. **(A)** Control (35S-GFP) under empty vector. **(B)** GhABP19-GFP fusion protein. **(C)** Plasmolyzed GhABP19-GFP fusion protein. Plasmolysis was induced with 0.8 M mannitol for 10 min. Scale bar represents 50 μm.

The subcellular localization of GhABP19 was experimentally determined using GhABP19-GFP fusion protein expressed in the *Arabidopsis* seedling root cells, the GFP fluorescence was observed in extracellular space by Confocal Laser Scanning Microscopy (Figure 6). The result from Figure 6B showed that GhABP19 was located in the cell wall or in the plasma membrane. The seedlings were treated with 0.8 M mannitol for 10 min to differentiate between the plasma membrane and cell wall location. After plasmolysis, the GFP fluorescence in Figure 6C revealed that GhABP19 was localized in the plasma membrane, which was consistent with the predictions conducted by TMHMM Server v 2.0^8^, WoLF PSORT^9^ and DeepLoc-1.0^10^.

### GhABP19 Overexpression in *Arabidopsis* in Response to *V. dahliae* and *F. oxysporum* in Soil

The presence and expression of *GhABP19* in hygromycin-resistant *Arabidopsis* lines were identified by genomic PCR analysis (data not shown). Homozygous transgenic (T3 generation) lines of L1, L3, and L4 were selected for subsequent experiments based on qRT-PCR analysis, the transcript levels were normalized relative to the line with the lowest transgenic expression (L10; Supplementary Figure S1B).

**FIGURE 7:**
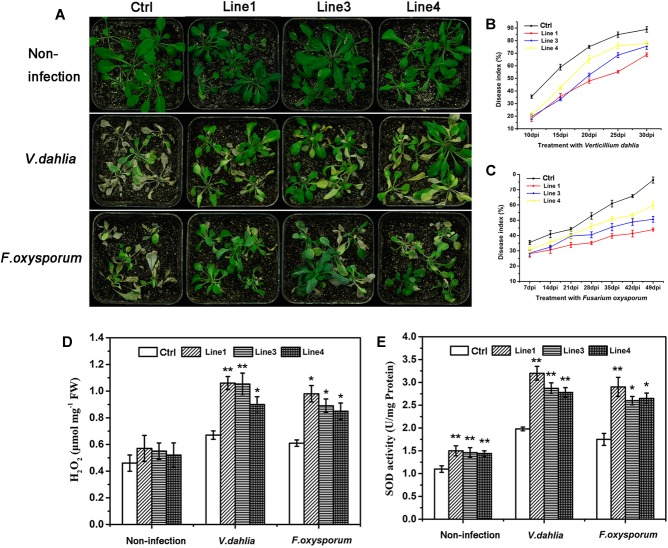
Resistance and analysis of H_2_O_2_ content, endogenous SOD activity in transgenic *Arabidopsis* plants. **(A)** Non-infected control (empty vector) and *GhABP19*-transgenic *Arabidopsis* plants, and *Arabidopsis* plants infected with *V. dahliae* and *F. oxysporum* in soil 30 and 49 dpi, respectively. **(B,C)** Plant disease indexes of control and transgenic *Arabidopsis* plants at the indicated days after inoculation with *V. dahliae* **(B)** and *F. oxysporum* **(C)**. Error bars represent the standard error of three biological replicates (*n* ≥ 30). **(D)** H_2_O_2_ content and **(E)** endogenous SOD activity in the leaves of control and *GhABP19*-transgenic *Arabidopsis* lines before and after inoculation with *V. dahliae* and *F. oxysporum* at 48 hpi. Error bars represent the standard error of three biological replicates (*n* ≥ 20). Asterisks indicate a significant difference compared with control (^∗^*P* < 0.05, ^∗∗^*P* < 0.01, Student’s *t*-test). FW, fresh weight; Ctrl, Control; Line 1, Line 3, Line 4, transgenic *Arabidopsis* lines.

To verify the antifungal activity of the *GhABP19* gene in plants, *GhABP19*-transgenic *Arabidopsis* plants were infected with *V. dahliae* and *F. oxysporum* in soil, respectively. For the fungus infection assay, 4-week-old transgenic and control *Arabidopsis* plants were infected by dipping the roots in fungal culture and co-cultivated in a growth chamber, in which non-infected plants served as a control. Disease incidence and severity were estimated using the PDI. For *V. dahliae* infection, clear differences were noted between wild-type and transgenic plants at 30 days post inoculation (dpi): wilting, yellowish color, and necrosis appeared on most leaf surfaces of control plants, whereas transgenic plants grew regularly, despite slight disease symptoms being visible on some leaves (Figure 7A). Plants of transgenic L1, L3, and L4 lines showed lower PDI (73.8 ± 1.1%, 75.5 ± 2.21%, and 77.8 ± 1.29%, respectively) compared with those of the control plants (85.9 ± 3.18%) at 30 dpi (Figure 7B). With regard to *F. oxysporum* infection, more than 70.4 ± 1.2% of the leaves of control plants were dead at 49 dpi. Compared with this result, symptoms detected on the older leaves and branches of transgenic plants were considerably milder (Figure 7A,C). These results are consistent with the antifungal activity of the GhABP19 protein and showed that the expression of *GhABP19* in *Arabidopsis* reduces the susceptibility of plants to pathogenic infection.

### Resistance of Transgenic *Arabidopsis* Seedlings to *F. oxysporum*

**FIGURE 8:**
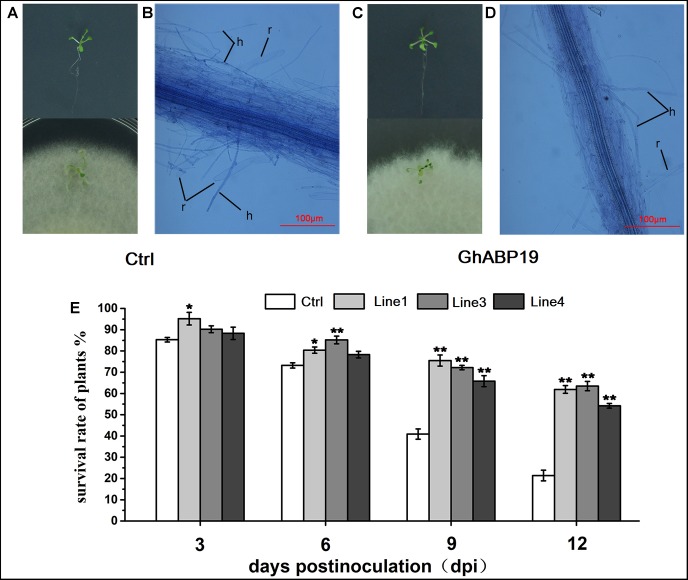
Infection assays with *F. oxysporum* on *Arabidopsis* seedlings and light microscopic observation of the *F. oxysporum* infection process on *Arabidopsis* seedlings roots. **(A)** Control (empty vector) *Arabidopsis* plants grow on MS plate before infection and 12 dpi with *F. oxysporum*. **(B)** Attachment and directed-growth of *F. oxysporum* hyphae over the root of control *Arabidopsis* plant. **(C)** *GhABP19* transgenic *Arabidopsis* grow on MS plate before infection and 12 dpi with *F. oxysporum*. **(D)** *F. oxysporum* growing on the root surface of *GhABP19* transgenic *Arabidopsis* plant without firm attachment. **(E)** Survival rate of control and transgenic *Arabidopsis* seedlings infected with *F. oxysporum.* Error bars represent the standard error of three biological replicates (*n* ≥ 20). Asterisks indicate a significant difference compared with control (^∗^*P* < 0.05, ^∗∗^*P* < 0.01, Student’s *t*-test). r, root; h, hyphae; Ctrl, Control; Line 1, Line 3, Line 4, transgenic *Arabidopsis* lines.

To substantiate the data obtained from infection experiments in soil, seedlings of control and *GhABP19-*transgenic *Arabidopsis* line with the highest *GhABP19* expression level (L1) were infected with *F. oxysporum* on agar plates to observe the penetration and development conditions of hyphae. Firstly, control and transgenic plants were transferred to agar plates inoculated with *F. oxysporum* mycelium and co-cultured in a growth chamber. Twelve days after infection, control *Arabidopsis* plants were severely infected with fungus (Figure 8A,B). They showed strong disease symptoms and 78.6 ± 2.5% died after infection (Figure 8E). However, transgenic plants only showed mild wilt (Figure 8C,D), and more than 54.2 ± 1.1% still grew normally (Figure 8E).

For microscopic observation of the *F. oxysporum* infection process, the infected roots were stained with trypan blue solution at 12 dpi. The roots were flushed with running tap water to remove most of the non-attached mycelium. Compared with the control roots, the hyphae on the root surface and mycelium inside the roots of transgenic *Arabidopsis* were remarkably reduced (Figure 8B,D). This indicated that both the penetration and development of the fungus in transgenic *Arabidopsis* roots were strongly inhibited. In conclusion, the microscopic observations supported the findings of the infection experiments in soil in that *GhABP19* inhibited fungal infections in transgenic *Arabidopsis* plants.

### *GhABP19* Silencing and Cotton Resistance to *V. dahliae* and *F. oxysporum*

**FIGURE 9:**
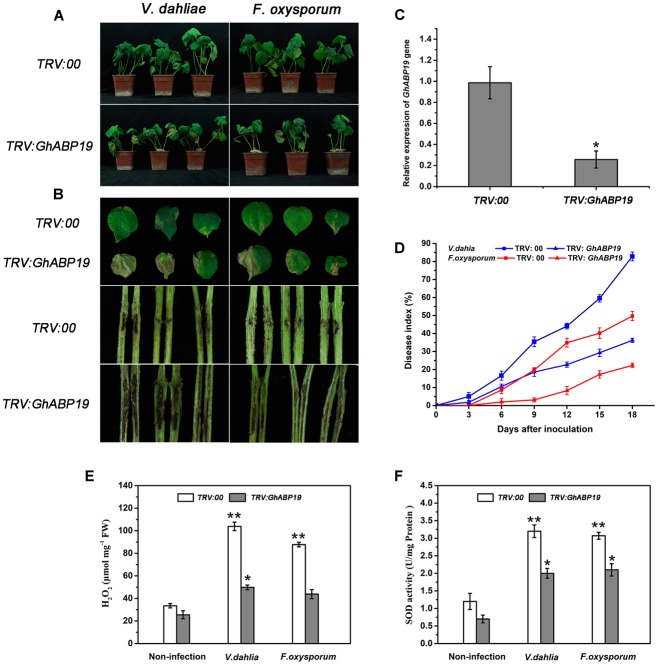
Susceptibility of *GhABP19*-silenced Zhongzhimian 2 cotton cultivar to *V. dahliae* and *F. oxysporum*. **(A)** Disease symptoms of control (*TRV:00*) and *GhABP19*-silenced (*TRV:GhABP19*) cotton plants infected by *V. dahliae* and *F. oxysporum* 18 dpi. **(B)** Representative leaves and vascular browning in stem of the control and *GhABP19*-silenced cotton plants infected by *V. dahliae* and *F. oxysporum* 18 dpi. **(C)** Mean expression levels of *GhABP19* gene analyzed by qPR-PCR. Total RNAs were extracted from the leaves of cotton plants after 2 weeks of VIGS, and the expression level of *GhABP19* in VIGS plants was compared with that of the control plants (*TRV:00*). Error bars represent the standard error of three biological replicates (*n* ≥ 20). Asterisks indicate a significant difference compared with control (^∗^*P* < 0.05, Student’s *t*-test). **(D)** Disease index of control and *GhABP19*-silenced cotton plants at the indicated days after inoculation with *V. dahliae* and *F. oxysporum*, respectively. Error bars represent the standard error of three biological replicates (*n* ≥ 30). **(E)** H_2_O_2_ content and **(F)** endogenous SOD activity in the leaves of control and *GhABP19*-silenced cotton plants before and after inoculation with *V. dahliae* and *F. oxysporum*, respectively. Error bars represent the standard error of three biological replicates (*n* ≥ 20). Asterisks indicate a significant difference compared with non-infection plants (^∗^*P* < 0.05, ^∗∗^*P* < 0.01, Student’s *t*-test). FW, fresh weight.

Virus-induced gene silencing is a powerful tool for determining the various functions of genes (Lou and Baldwin, 2006; Gao et al., 2011; Mejía-Teniente et al., 2015). To clarify the role of *GhABP19* in the defense response of cotton against *V. dahliae* and *F. oxysporum*, we used *Agrobacterium-*mediated VIGS to generate *GhABP19*-silenced Zhongzhimian 2 cotton cultivar (*TRV:GhABP19*) and control plants (*TRV:00*). Further, the cotton gene *GhCLA1*, that is involved in chloroplast development and which needed to be silenced, would be a visual marker to monitor VIGS efficiency (Gao et al., 2011). The silencing of *GhABP19* and *GhCLA1* was confirmed by semi-quantitative RT-PCR and qRT-PCR analysis after 2 weeks of VIGS (Figure 9C and Supplementary Figure S2). The results showed that *GhABP19* was successfully knocked down.

To investigate the role of *GhABP19* in cotton, control and *GhABP19-*silenced Zhongzhimian 2 were challenged with spore suspensions of *V. dahliae* and *F. oxysporum* by syringe inoculation. As results, yellow wilted leaves appeared 6 days after inoculation in *GhABP19-*silenced cotton. After 18 days, there were severe necrosis symptoms in the leaves of *GhABP19-*silenced plants (Figure 9A,B). The vertical vascular tissue in *GhABP19-*silenced plant stems showed more serious discoloration than that of control plants (Figure 9B). The plant disease index of control plants was much higher than that of *GhABP19-*silenced plants after inoculation with *V. dahliae* and *F. oxysporum*, respectively (Figure 9D). The disease symptoms of cotton plants suggested that silencing *GhABP19* increased plants’ susceptibility to *V. dahliae* and *F. oxysporum* infection.

### H_2_O_2_ Accumulation and Endogenous SOD Activity in Transgenic *Arabidopsis* Lines and *GhABP19*-Silenced Cotton Plants

Previous studies have shown that several GLPs exhibit SOD activity, which leads to H_2_O_2_ production and is often correlated with plant defense responses (Sultana et al., 2016; Zhang et al., 2017, 2018). In our study, the recombinant GhABP19 protein exhibited SOD activity *in vitro*. To confirm whether the enhanced disease resistance in *GhABP19*-transgenic *Arabidopsis* plants is related to H_2_O_2_ accumulation resulting from endogenous SOD activity of *GhABP19*, we analyzed the H_2_O_2_ content and endogenous SOD activity in transgenic *Arabidopsis* plants. After inoculation with *V. dahliae* and *F. oxysporum*, respectively, all the *GhABP19* transgenic lines showed significantly higher H_2_O_2_ levels compared to those in control plants at 48 hpi (Figure 7D). Moreover, the endogenous SOD activity was determined in the same plants used for H_2_O_2_ quantification. As shown in Figure 7E, endogenous SOD activity in transgenic *Arabidopsis* plants was elevated before and after pathogen inoculation, respectively.

In addition, the H_2_O_2_ contents and SOD activity of *TRV:00* and *TRV:GhABP19* plants were measured after inoculation with fungi. The levels of H_2_O_2_ and SOD activity in non-infected *TRV:00* and *TRV:GhABP19* exhibited little difference. However, endogenous H_2_O_2_ contents and SOD activity increased significantly in both *TRV:00* and *TRV:GhABP19* after pathogen inoculation (Figure 9E,F), suggesting that the silence of *GhABP19* is responsible for reducing H_2_O_2_ production and SOD activity.

### The Expression of Defense-Related Genes Involved in JA Signaling Pathway in Transgenic *Arabidopsis* Lines and *GhABP19*-Silenced Cotton Plants

**FIGURE 10:**
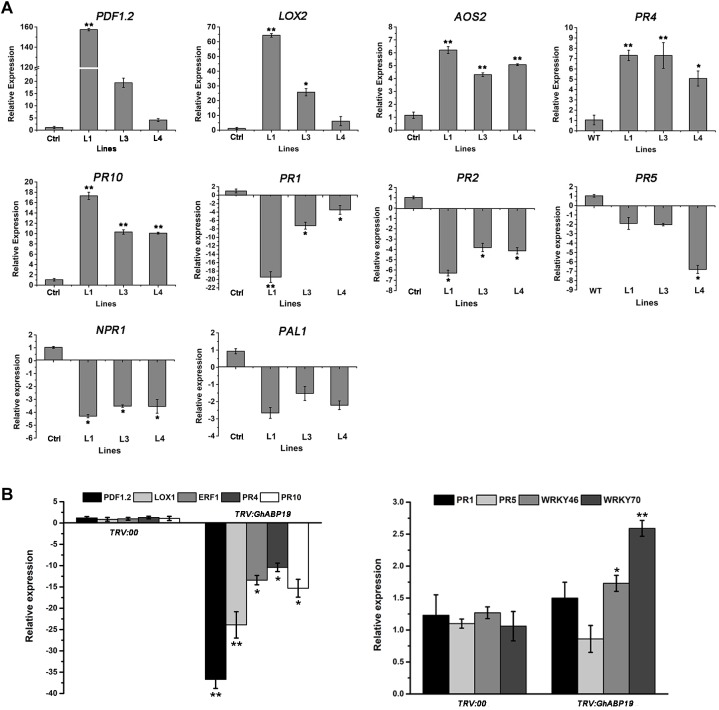
qRT-PCR analyses of JA- and SA-related genes. **(A)** The relative expression of genes in non-inoculated control and *GhABP19*-transgenic *Arabidopsis* lines. *AtEF1α* was used as an internal standard. **(B)** The relative expression of genes in non-inoculated *TRV:00* and *TRV:GhABP19* cotton plants. *GhUBQ7* was used as an internal standard. The fold down-regulation was presented as calculated by the formula –1/normalized gene expression value. Data were collected from three independent biological samples with three technical replicates. Error bars represent the standard error. Asterisks indicate a significant difference compared with control (^∗^*P* < 0.05, ^∗∗^*P* < 0.01, Student’s *t*-test). Ctrl, control; L1, L3, L4, transgenic *Arabidopsis* lines.

Based on the observation that *GhABP19* expression was induced by JA but depressed by SA treatments, we examined whether the resistance conferred by *GhABP19* observed in this study was correlated with the induction of JA and/or SA endogenous defense signaling pathways. The expression patterns of some defense-related marker genes involved in the JA (*PDF1.2*, *LOX2*, *AOS2*, *PR4*, and *PR10*) and SA pathway (*PR-1*, *PR-2*, *PR-5*, *NPR1*, and *PAL1*) were detected in non-inoculated *GhABP19*-transgenic *Arabidopsis* lines and control plants by qRT-PCR. Even without pathogen infection, JA pathway–related *AOS2*, *PR4*, and *PR10* were strongly induced in all *GhABP19*-transgenic lines compared with the control plants (Figure 10A). In contrast, the expression levels of *PR-1*, *PR-2*, and *NPR1*, which are associated with the SA pathway, were significantly downregulated in *GhABP19*-transgenic *Arabidopsis* lines, and there was no difference in the expression of *PR-5* and *PAL1* genes (Figure 10A).

We also analyzed the expression levels of several well-characterized JA- and SA-related defense genes in non-inoculated *GhABP1*9-silenced cotton plants. The expression levels of *PDF1.2*, *LOX1*, *ERF1*, *PR4*, and *PR10*, which are involved in JA-related defense responses, were significantly down-regulated by *GhABP1*9 suppression in cotton (Figure 10B). Silencing of *GhABP1*9 did not alter the transcripts of SA-related genes *PR1* and *PR5*, however, the expression of *WRKY46* and *WRKY70* in SA signal pathway were increased in the *GhABP1*9-silenced plants (Figure 10B).

**Table 2:** Pathogenesis-related elements in the promoter of GhABP19.

Gene	Sequence	References
(a) GT-1 BOX		
Gm SCaM-4	GAAAAA	Park et al., 2004
ABP19	GAAAAA (990/561)	This paper
(b) W-box		
TGAG-containing W box element		
Pc WRKY1	TGAC	Eulgem et al., 1999
ABP19	TGAC (1410/165)	This paper
ELER (elicitor responsive element)		
Pc PR1	TTGACC	Eulgem et al., 1999
ABP19	TTGACC (757), TTGAC (683)	This paper
(c) SEBF (silencing element binding factor)		
St PR-10a	CTGTCAC	Boyle and Brisson, 2001
ABP19	TTGTCAC (29)	This paper

### Analysis of *cis*-Elements in *GhABP19* Promoter

To analyze the pathogen resistance–related *cis*-elements in *GhABP19* promoter, the 1500 bp promoter sequence from the transcription start site of *GhABP19* was predicted by PLACE. The putative results revealed that several regions might be important for the pathogen resistance conferred by the gene. Three types of pathogen resistance-related elements were included in the *GhABP19* promoter (Table 2 and Supplementary Figure S3). The GT-1-box in the promoter of the *Glycine max SCaM-4*gene plays a role in pathogen- and salt-induced *SCaM-4* gene expression (Park et al., 2004). Two sequences similar to the GT-1-box (GAAAAA) existed at positions -990 and -561 in *GhABP19*, respectively. Furthermore, WRKY proteins bind *in vitro* to functionally define the elicitor-response elements of the W-box elements [(T)TGAC(C)] present in *PR1* promoters (Eulgem et al., 1999). We found four W-box elements in the studied promoter, including two TGAG-containing W-box elements (TGAC) at positions -1410 and -165 and two elicitor responsive elements [ELER, TTGAC(C)] at positions -757 and -683, respectively. In addition, the binding site of the potato silencing element-binding factor (CTGTCAC) was found in the promoter of the pathogen resistance–related gene *PR-10a*(Boyle and Brisson, 2001), and a related element was found at position -29 in *GhABP19*. These three kinds of *cis*-elements might be involved in the fungal resistance of *GhABP19*. However, further studies are needed to investigate their specific functions.

## Discussion

Germin-like proteins (GLPs) are plant glycoproteins and a crucial component of plant basal host resistance (Zimmermann, 2006). In the present study, we characterized *GhABP19*, a novel GLP gene isolated from cotton and provided evidence for its role in the regulation of resistance to verticillium and fusarium wilt in plants.

Plant GLPs have been classified into five subfamilies (Carter and Thornburg, 2000), GhABP19 belongs to the GLP subfamily 3 and shares a common characteristic with other members of this group: they highly abundant in young leaves, less abundant in stems, and absent in roots (Figure 2, 3A; Heintzen et al., 1994; Ono et al., 1996; Vallelian-Bindschedler et al., 1998; Fan et al., 2005).

The general structure of GhABP19 is consistent with the typical organization of germins and GLPs (Figure 1B). In most of the GLP family members, box-A consists of a consensus sequence (QDFCVAD) (Bernier and Berna, 2001). However, GhABP19 is predicted to possess a sequence variation, as glutamine (Q) and alanine (A) are replaced by alanine (A) and glycine (G), respectively. In addition, a cysteine (C) residue at position 62 is included in GhABP19 box-A, which is followed by a second cysteine (C) at position 77, and could form an internal disulfide bridge of the extracellular domain (Woo et al., 2000). Box-B(G–P-H-HPGASEXXXXX-G), corresponding to amino acid residues from 132 to 151, is conserved at the inner position of the full-length sequence. Box-C is GXXHFQ-N-G, where X corresponds to any hydrophobic amino acid residue. There are two histidine (H) and one glutamate (E) residues in box-B, and box-C contains the third histidine residue, which in germins is involved in heavy metal ion-binding (Gane et al., 1998) and considered to be the ligand-binding conserved sequence in the auxin-binding protein/germin class (Dunwell and Gane, 1998; Woo et al., 1998). The RGD-like tripeptide motif sequence, detected in over 50% of GLPs characteristically involved in protein–protein interactions, was also present (Figure 1B; Labour et al., 1999). In animal cells, these tripeptide domains are found in cell adhesion proteins from the extracellular matrix (such as vitronectin and fibronectin) that interact with transmembrane proteins called integrins (Labour et al., 1999).

The results of bioinformatic analysis and subcellular localization experiments showed that GhABP19, is a transmembrane protein distributed at plasma membrane or cell wall (Figure 6B). After plasmolysis, GhABP19 was confirmed to locate in the plasma membrane (Figure 6C), which was consistent with the localization of several GLPs in *Arachis hypogaea* (Wang et al., 2013).

Previous studies have shown that GLP genes might be essential for plant general resistance to biotic stress; the expression of GLPs is differentially regulated in response to pathogen infection in different plant species (Breen and Bellgard, 2010). For instance, GLPs in *B. vulgaris*, *Brassica napus*, *A. hypogaea*, and *L. regale* show a broad spectrum of defensive activities in host–pathogen interactions (Knecht et al., 2010; Rietz et al., 2012; Wang et al., 2013; Zhang et al., 2017). In this study, we used the resistant Zhongzhimian 2 and the susceptible Xinluzao 33 cotton cultivars to investigate a possible role of *GhABP19* gene in response to *V. dahliae* and *F. oxysporum* infection. The result reported in the literature indicate that the susceptible cultivar exhibited a little more accumulated increased fungal biomass when compared with the resistant cultivar in both *V. dahliae* and *F. oxysporum*; this may be partially reflected in the difference between resistant and susceptible varieties (Liu et al., 2017a). Our data showed that the *GhABP19* gene in two non-treated cotton cultivars both showed high transcript abundance in the leaf tissue, indicating conserved gene regulation under normal growth conditions (Figure 3A). After inoculation with *V. dahliae* and *F. oxysporum*, there was first an increase in *GhABP19* expression, followed by its decrease in both cotton cultivars, and the expression levels of *GhABP19* in Zhongzhimian 2 were a bit higher than that in Xinluzao 33 (Figure 3B,C), suggesting a possible role in plant basal resistance. Notably, an obvious difference existed between the expression levels of *GhABP19* induced by *V. dahliae* and *F. oxysporum*, respectively. Take Zhongzhimian 2 for example, the expression of *GhABP19* was up-regulated by about 20-fold after inoculation with *V. dahliae* at 0.5 hpi, but it was only up-regulated by 2.2-fold when treated with *F. oxysporum* at the same hpi. Based on this result, we predicted that *GhABP19* is considerably more instrumental in enhancing the resistance of cotton to *V. dahliae* infection than it is to *F. oxysporum* infection.

*Arabidopsis* transgenic technology and silencing the endogenous genes through VIGS are two convenient methods for gene function characterization, and these approaches were employed to determine the role of *GhABP19* in *Arabidopsis* and cotton plants, respectively. The *GhABP19-*transgenic *Arabidopsis* lines were infected with *V. dahliae* and *F. oxysporum* to assess their resistance responses, respectively (Figure 7). The results revealed that overexpression of *GhABP19* in *Arabidopsis* plants enhanced their disease resistance, reducing chlorosis and death of transgenic plants. Furthermore, microscopic observation of *Arabidopsis* roots infected with *F. oxysporum* provided evidence for the accumulation of *GhABP19*, which could prevent the penetration and development of the fungus (Figure 8). In *GhABP19*-silenced cotton plants, *GhABP19* silencing reduced *V. dahliae* and *F. oxysporum* resistance of cotton seedlings, as determined by pathogen inoculation assays (Figure 9). Furthermore, the necrosis of leaves and the discoloration of the vascular network in *GhABP19*-silenced cotton plants caused by *V. dahliae* are more serious than that caused by *F. oxysporum* (Figure 9).

Reactive oxygen species, especially H_2_O_2_, are generated in pathogen-infected plants. H_2_O_2_ plays various roles in host–pathogen interactions, and it is suggested as an antimicrobial agent in plant defense responses (Walters, 2003). It is further involved in different signaling pathways associated with defense mechanisms, such as triggering of the hypersensitivity response, accumulation of phytoalexins, and activation of many other defense-response genes (Shetty et al., 2008). However, overaccumulation of reactive oxygen species in plant cells damages biomolecular components, such as membrane lipids, nucleic acids, chloroplast pigments, and proteins (Verma and Dubey, 2003). To overcome oxidative damage, plants have an antioxidant defense system comprising various enzymes, for example, SOD can remove, neutralize, and scavenge reactive oxygen species by converting O_2_^-^ to H_2_O_2_ and O_2_ (Shah et al., 2001).

In our study, the recombinant GhABP19 protein exhibited SOD activity (Figure 5), and hyphal extension inhibition assays provided evidence of direct antifungal activity of GhABP19 on various pathogens (Table 1). This suggests that the resistance of the GhABP19 protein observed in the *in vivo* experiment can be attributed to the direct effect of SOD activity. Moreover, the H_2_O_2_ content and endogenous SOD activity were measured in *GhABP19-*transgenic *Arabidopsis* plants and *GhABP19*-silenced cotton plants after *V. dahliae* and *F. oxysporum* infection (Figure 7D,E, 9E,F). Notably, H_2_O_2_ content and SOD activity were elevated in *GhABP19-*transgenic *Arabidopsis* plants compared to control plants; and as expected, silencing of *GhABP19* decreased the accumulation of H_2_O_2_ and the activity of endogenous SOD enzyme. One possible explanation for the role of *GhABP19* in plant fungal resistance could therefore be the accumulation of H_2_O_2_ produced by SOD activity.

Although the increase in H_2_O_2_ might be a GhABP19-mediated defense response to fungal infection, it remains unclear whether this increase leads to the activation of any signaling pathways. JA and SA are important phytohormones in regulating plant disease resistance (Thatcher et al., 2005). Given the *GhABP19* expression was induced after treatment with exogenous JA but suppressed by SA treatment (Figure 3D,E), we hypothesized that *GhABP19* likely involved in phytohormone signaling regulation. Here, the expression of some genes involved in the SA and JA pathways was analyzed. In *Arabidopsis* plants, the overexpression of *GhABP19* activated the transcript levels for *AOS2*, *PR4*, and *PR10*, which depend on the JA signaling pathway but decreased the expression levels of selected genes associated with the SA pathway, such as *PR-1*, *PR-2*, and *NPR1* (Figure 10A). The activation of JA-related genes in the transgenic *Arabidopsis* lines even in the absence of pathogens better protected the plant. Moreover, *PDF1.2*, *LOX1*, *ERF1*, *PR4*, and *PR10* in JA signal pathway were suppressed in non-inoculated *GhABP1*9-silenced cotton plants, and minimal changes were identified in the levels of expression of genes associated with the SA-signal pathway, such as *PR1* and *PR5* (Figure 10B). Meanwhile, the expression levels of *WRKY46* and *WRKY70*, which are involved in SA-related defense responses, were up-regulated by *GhABP19* suppression in cotton without pathogen infection. These results revealed that several defense-related genes in JA signal pathway were suppressed by silencing *GhABP19*. GhABP19 is functional as a JA-signaling component in plant resistance mechanisms by specifically regulating the expression of a set of plant defense-related genes before pathogen infection.

## Conclusion

The functional analysis of GhABP19, a GLP protein with SOD activity, revealed a potential role in plant disease resistance. Our findings show that ectopic overexpression of *GhABP19* protected transgenic *Arabidopsis* against *V. dahliae* and *F. oxysporum* infection. Conversely, resistance to *V. dahliae* and *F. oxysporum* was decreased by silencing *GhABP19* in cotton. The disease defensive function of GhABP19 was exerted by generating H_2_O_2_ via its SOD activity, as well as upregulating the transcription levels of several defense-related genes involved in JA pathways. These results highlight the potential application of GhABP19 in biotechnology and provide a basis for developing strategies to improve disease resistance in cotton plants. Moreover, several defense-related *cis*-elements are present in the promoter of GhABP19 (Supplementary Figure S3 and Table 2), but their exact functions are not yet known and need to be determined in the future.

## Author Contributions

YP, YH and FL conceived and designed the study. YP conducted most of the experiments, analyzed the data, and wrote the manuscript. XL, YZ, XG, and YJ provided technical assistance to YP. YS and NL provided analysis tools. All authors reviewed and revised the manuscript and figures.

## Conflict of Interest Statement

The authors declare that the research was conducted in the absence of any commercial or financial relationships that could be construed as a potential conflict of interest.

## Footnotes

1 https://www.ncbi.nlm.nih.gov/orffinder/

2 http://www.cbs.dtu.dk/services/SignalP/

3 http://www.expasy.org/tools/protparam.html

4 https://www.ebi.ac.uk/Tools/msa/clustalo/

5 https://www.swissmodel.expasy.org/

6 http://www.sbg.bio.ic.ac.uk/~{}ezmol/

7 http://www.dna.affrc.go.jp/PLACE/

8 http://www.cbs.dtu.dk/services/TMHMM/

9 https://wolfpsort.hgc.jp/

10 http://www.cbs.dtu.dk/services/DeepLoc/
