# Supplementary material for: GhABP19, a Novel Germin-Like Protein From Gossypium hirsutum, Plays an Important Role in the Regulation of Resistance to Verticillium and Fusarium Wilt Pathogens
Source: Front Plant Sci. 2019 May 8;10:583. doi: 10.3389/fpls.2019.00583 (PMC6517559; doi:10.3389/fpls.2019.00583)
Supplement: Supplementary file 3 [file Image_1.pdf]

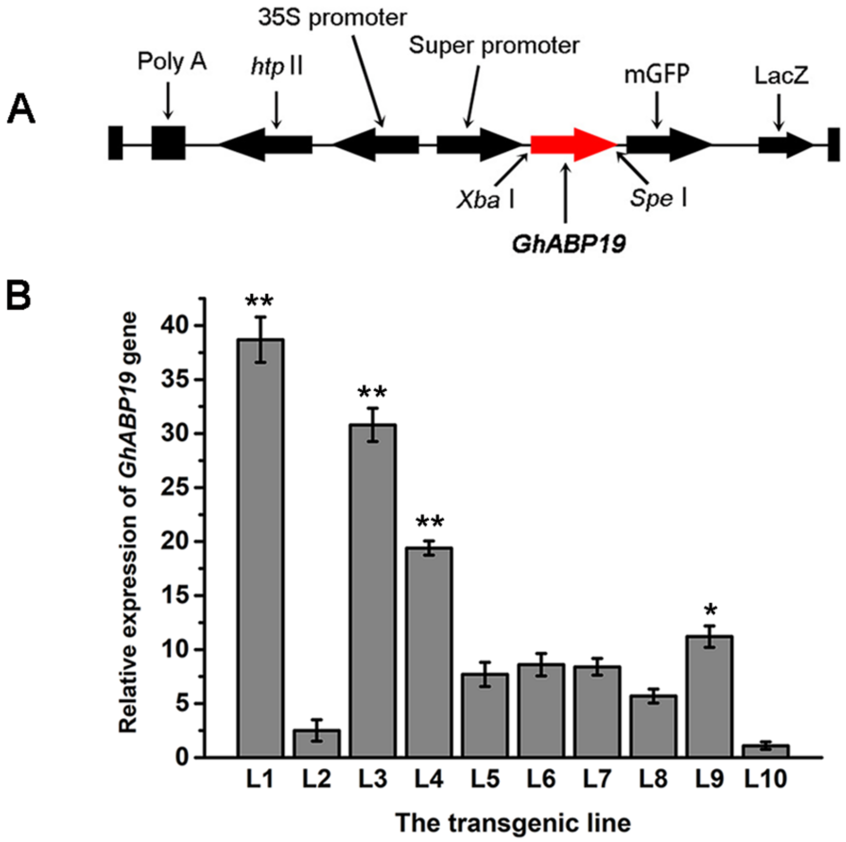

Supplementary Figure 1. Genetic transformation of *Arabidposis* with *GhABP19* and identification of transgenic lines. **(A)** Outline of the Super-pCAMBIA1300 transformation vector with GhABP19 under the control of CaMV 35S promoter. **(B)** RT-qPCR analysis. Transcript levels of *GhABP19* in transgenic *Arabidopsis* lines relative to the line with the lowest transgene expression (L10). All measurements were normalized with the *EF1a* transcript level. Data were collected from three independent biological samples per treatment and three technical replicates per samples. Error bars represent standard error. Asterisks indicate a significant difference (*P < 0.05 and **P <0.01, Student's *t* test).
