# Supplementary material for: GhABP19, a Novel Germin-Like Protein From Gossypium hirsutum, Plays an Important Role in the Regulation of Resistance to Verticillium and Fusarium Wilt Pathogens
Source: Front Plant Sci. 2019 May 8;10:583. doi: 10.3389/fpls.2019.00583 (PMC6517559; doi:10.3389/fpls.2019.00583)
Supplement: Supplementary file 4 [file Image_2.pdf]

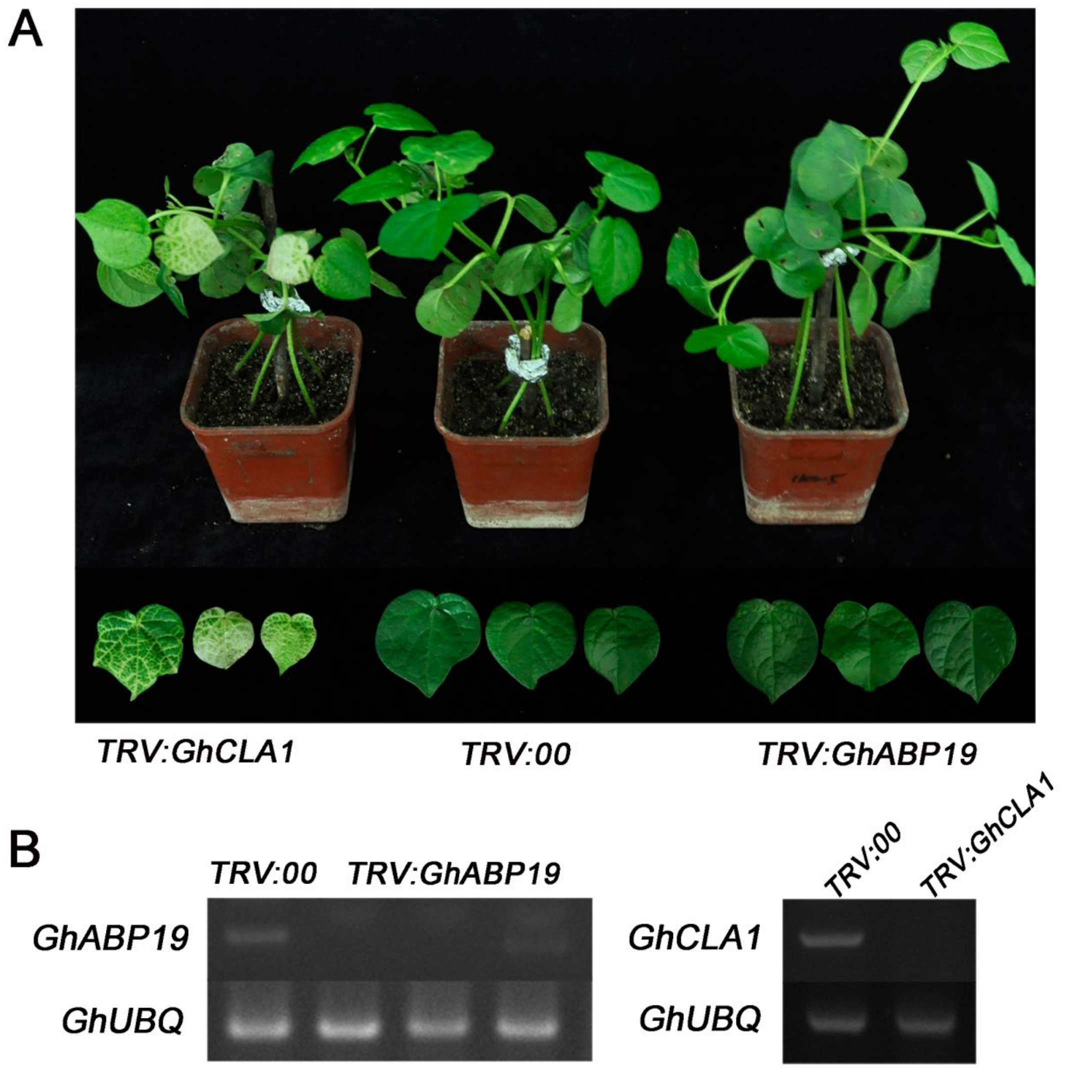

Supplementary Figure 2. VIGS-mediated gene silencing in Zhongzhiian 2 cotton cultivar. **(A)** The phenotypes of *GhCLA1*-silenced, control and *GhABP19*-silenced cotton plants. The top panel shows the plant phenotypes, the bottom panel shows the leaf phenotypes. **(B)** The expression of *GhABP19* and *GhCLA1* in the control and silenced cotton were analyzed by semi-quantitative RT-PCR. *GhUBQ* was used as a reference gene. The experiments were carried out with three repeats and showed similar results.
