# Supplementary material for: GhABP19, a Novel Germin-Like Protein From Gossypium hirsutum, Plays an Important Role in the Regulation of Resistance to Verticillium and Fusarium Wilt Pathogens
Source: Front Plant Sci. 2019 May 8;10:583. doi: 10.3389/fpls.2019.00583 (PMC6517559; doi:10.3389/fpls.2019.00583)
Supplement: Supplementary file 5 [file Image_3.pdf]

```
TAACAAGGGAATCCGATTTTTCCAGCTAGAAATTGAATGGCTATTGGCTT  1451
GGCCCTTACTCTTCTGTCATTGTCAAACCTTATCTTTGCTGACACTTACA  1401
                                       b-1
CTTTTAGGGAACTGGCTCCTACCCTTCATGCATTAGGAACCGTTAGCTTT  1351
ACTTGTAGATAGATTAGATATTTACCTATCTCTGCTAGAAGATTCTTATT  1301
TTGGTTCCTTGATTCTTGTTGGCTTCAAGTTTGAGACAAATAAGCCATTA  1251
ACTCGTACCCTGAAGAAGGAGAATTACTCGAACTTAATCAGAAGTAAATC  1201
AGATATACTAGATAAAACATTTTTGCAAACAATTCTTATCTAGCCACTTT  1151
GTGTGAATATATATATATTCCTTGCTTATTGTACTTGGAAGTAACTGCAG  1101
CTGAATGGTGAATAAATTGTTTCCACAGTTTCTAGTAGGTTCAAGTAAGG  1051
GCAATCAGATGCTCAGGGCAGAGATAAGATAACCCTTGCTAGGTCTTCCT  1001
GGGACCACGAAAAATGCTTATCAACTAAACTTCCAAATTTACAGTTTTGA   951
         a-1
TATTTTACATTTTCCTAAGGATCTTGCATTTTCATGAACGAAAGTTTGAG   901
AGACAACTTTTTTACCCTTCTGAACGTAATATAGATATGCCTTGTAAGAC   851
AGGGAATTAGCAAGCTCCACATATTTTTTTCCCATGGAGATAAATCACT    801
TGCAGAGTCTCTTCTTATTAGCTGTCAAAAAATATCTTCTATTGACCTAA   751
                                          b-3
CTTACTCCCTTCTAACGTGCTGTGTAGCCAATGGACCACTTTTTGATATA   701
ACTTTCATACTCTTCTTGACAATGATCTCGGTTTTCCATGTTTAATTATG   651
          b-4
CCCCTTAATAACGGGTTATGCTGTTTGTATTATTGTAAATGTATATAGAA   601
AACTTTGAATTGATGTTTGTAAAGATGTGATTGCATTGAAAAATACATAA   551
                                     a-2
TATCACCTTCAAAGTCTCCCTTAAATTATGCAGAATTAGTAGTGTTACAA   501
TCAATCTATCATCTCTAATACCAACTGTTTGATGGATACACCTACTGAAC   451
GTTTCATTTAAGGAAGAGAAGCCTTTGCTTTCATCGAAACATTTATGATA   401
CGCATGCACTCAATGAAAATTCCTGTTTCCTTAATGCTAGCAGAAAGTCC   351
AAGGTTGCATATATCAGGGATTTAAGTAGACATTTACCACAATTAACTAT   301
GATGGCATCTAAATCCTGGAATATATATCAAAAGCTGATGCTAGATTATA   251
AGTCATTTTTCTGAATGTATAGATGCATGGCCATGGCTGAAACCTGATTC   201
TCCTTGTAAAATGATAAACAGTAAAAACATATGTGACATGAGAAATGAAA   151
                                 b-2
ATTACCTGCAACAATTGGATGCAAAAGATATTTTTTTCCAATCCAAAAGA   101
GTATTCTTGTTGTTTTCTGAGAAAAATTTTCCTCTAATTCACATGCATCA    51
CTGCTTCAAAATCTGTCGCTTGTCACATAGGGAATCAGTCTCATCATCAT     1
                   c-1
```

Supplementary Figure 3. A sequence of 1500 bp promoter of *GhABP19* and putative regulative elements. a, GT-1 box; b, W-box and c, silencing element binding factor. The numbers correspond to those in Table 2.
